# Plastome phylogenomics and characterization of rare genomic changes as taxonomic markers in plastome groups 1 and 2 Poeae (Pooideae; Poaceae)

**DOI:** 10.7717/peerj.6959

**Published:** 2019-06-03

**Authors:** Lauren M. Orton, Sean V. Burke, Melvin R. Duvall

**Affiliations:** 1 Plant Molecular and Bioinformatics Center, Biological Sciences, Northern Illinois University, DeKalb, IL, United States of America; 2 Center for Translational Data Science, University of Chicago, Chicago, IL, United States of America

**Keywords:** Poeae, Chloroplast, Phylogenomics, Taxonomic markers, Rare genomic changes, Intrastrand deletion

## Abstract

A phylogenomic analysis of 42 complete plastid genomes (plastomes), including 16 that were newly sequenced, was conducted. Plastomes were sampled from 19 subtribes of Pooideae, to investigate relationships within and between Chloroplast Group 1 (Aveneae) and Group 2 (Poeae) species. Two data partitions: complete plastomes, and a combined plastome and rare genomic change (RGC) data matrix, were analyzed. Overall, 156 non-ambiguous RGC were identified, of which homology was inferred for 38 RGC. Among the 38 RGC identified, six were synapomorphic among the Group 1 subtribes: Aveninae, Agrostidinae, and Anthoxanthinae, (Phalaridinae + Torreyochloinae), and 27 were synapomorphic among the Group 2 subtribes: Loliinae, (Ammochloinae + Parapholiinae + Dactylidinae), Parapholiinae, Dactylidinae, Poinae, and Coleanthinae. Four RGC were determined to be homoplasious in Groups 1 and 2. Two other RGC originated through intrastrand deletion events. The remaining RGC events likely originated through recombination given their size and lack of sequence evidence for other types of mutations. This study also determined that relationships between taxa, even those only weakly supported in previous studies, could be inferred with strong support when utilizing complete plastomes.

## Introduction

As one of the economically significant lineages in Poaceae, Pooideae is the largest of the 12 grass subfamilies and has been particularly studied with regard to crop production. Over 70% of the human population depends on wheat, oat, barley, or rye as a staple in daily dietary nutrition (USDA, 2016). In 2016, global wheat yields exceeded 754 million metric tons, 22.5 million metric tons of oats were harvested, global barley production was approximately 150 million metric tons (USDA, 2017b), and rye accounted for an estimated 13 million metric tons (FAOSTAT, 2019).

Pooideae comprise over 4,000 species, 200 genera, and 14 tribes of cool season, C_3_ photosynthetic grasses (Soreng et al., 2015; Saarela et al., 2015). Distribution of the Pooideae subfamily of grasses naturally ranges from Arctic North America to South America, Europe, and into other temperate climates through introduction (Soreng et al., 2015). In spite of the lack of robust morphological synapomorphies, Pooideae exhibit some morphological trends (Kellogg, 2015): ligules are typically membranous, a fringed membrane, or a fringe of hairs, and lodicules are also membranous. The subsidiary cells generally have parallel sides, and veins in the leaf are widely spaced, as is typical in C_3_ species (Kellogg, 2015). Extensive research has been conducted to better understand the phylogenetics of this subfamily (Soreng et al., 2017; Saarela et al., 2017).

Within Pooideae, the tribe Poeae is of particular interest. The Poeae is the largest tribe of grasses with over 2,800 species and 121 genera (Soreng et al., 2015; Soreng et al., 2017). Many common lawn, pasture, and crop grasses are members of this group. Poeae grasses are distributed mainly across the Western Hemisphere and Eurasia, although many genera maintain broad distributions including areas of Africa, Asia, and Australia (Soreng et al., 2017). A division exists in the Poeae in which phylogenetic analyses of plastid loci cluster taxa into either “Chloroplast Group 1” (Aveneae-type, containing 41 genera) or “Chloroplast Group 2” (Poeae-type, containing 80 genera). In this paper, these clade names are shortened to the capitalized “Group 1 and 2.” This division was first identified by Soreng, Davis & Doyle (1990) on the basis of restriction site variation in chloroplast DNA, and substantiated in later publications of plastid characters (Davis & Soreng, 1993; Soreng & Davis, 1998; Soreng & Davis, 2000; Döring et al., 2007; Winterfeld, Doring & Röser, 2009; Saarela et al., 2015; Saarela et al., 2017; Saarela et al., 2018; Hodkinson, 2018). Aveneae sensu Dumortier & Charles (1824) was once recognized as a tribe, but later absorbed into the Poeae (Tzvelev, 1989; Soreng et al., 2015). In spite of considerable research, no morphological synapomorphies have been reported for Group 1 or 2 (e.g.,  Kellogg, 2015; Saarela et al., 2017). Neither is there a clear biogeographical basis for separation of these taxa (Soreng et al., 2015; Soreng et al., 2017). This same taxonomic division does not exist as clearly in analyses conducted with nuclear sequence data (Quintanar, Castroviejo & Catalán, 2007; Schneider et al., 2009; Hochbach, Schneider & Röser, 2015; Saarela et al., 2017). In earlier multi-gene studies, the fundamental diagnostic characters for Groups 1 and 2 appeared to be exclusively selected plastid loci (Soreng et al., 2015; used two plastid genes, *matK* and *ndhF* for analysis); (Saarela et al., 2017; used five plastid sequence regions and nrDNA: ITS and ETS). Complete plastomes infer the same two monophyletic groups, although taxonomic sampling is somewhat limited (Saarela et al., 2015; with 21 Poeae species in 18 genera), (Saarela et al., 2018; with 29 species in 24 genera).

In recent work, Agrostidinae, a Group 1 taxon, has been variously redefined at least partly in an effort to make the subtribe monophyletic. Kellogg (2015) recognized 21 genera, but Soreng et al. (2017) constrained it to 11. In part, these differing circumscriptions reflect paraphyly with other Group 1 taxa and the classification schemes used to accommodate differing phylogenetic results. Within Agrostidinae the affinities of the *Gastridium* + *Triplachne* clade are also uncertain, partly because of low support values in phylogenetic topologies inferred from multi-locus data (Saarela et al., 2017). The increase in support generally seen in plastome phylogenomic analyses of grasses could potentially address this uncertainty. The monophyly of *Polypogon* has also been questioned. Saarela et al. (2017) found *Polypogon* to be nonmonophyletic in nrDNA and plastid trees, and Soreng et al. (2017) indicated that the genus is reticulate. In Group 2, the relationships of Loliinae are also unclear partly because certain genera, such as *Castellia* and *Ammochloa*, have not been included in contemporary phylogenies. However, when using only a small portion of the genome, an incomplete picture is created and lack of phylogenetic information may impact the analysis (Burke, Grennan & Duvall, 2012; Burke et al., 2016a; Burke et al., 2016b; Saarela et al., 2015; Saarela et al., 2017; Saarela et al., 2018).

Plastid genes are quite conserved, however intergenic spacers (IGS) are less so, and are of greater potential phylogenetic utility. IGS regions are more likely to develop insertion/deletion mutations (indels), other microstructural changes (MC), or rare genomic changes (RGC) than coding sequences (CDS; Orton et al., 2017). The two classes of mutations, RGC and MC, are distinguished by mutational mechanism, size, and frequency. Most RGC are the result of nonreciprocal site-specific recombinations that cause large indels (>50 bp) and which occur infrequently. By contrast, MC are often due to slipped-strand mispairings or other interactions between repeated sequences affecting shorter regions (<50 bp), and are also more likely to be masked by succeeding mutation events because they occur with higher frequency (Graur, Sater & Cooper, 2016; Orton et al., 2017). In this study, the analysis of RGC additionally allows us to survey plastid characters less likely to be skewed by positive selection in coding sequences, especially those most commonly used in phylogenetic studies (Burke et al., 2016a; Burke et al., 2016b; Piot et al., 2018; Saarela et al., 2018). Identifying RGC, as defined by Rokas & Holland (2000), Jones, Burke & Duvall (2014), Duvall et al. (2016), Orton et al. (2017), may offer insights into how such unique mutations occur and persist. Given the diversity of species represented in this project, microstructural changes (MC) would likely fail to produce useful and inferential data because of saturation causing difficulties in homology assessment (Orton et al., 2017). However, RGC are more readily identified and interpreted in diverse groups of species due to their larger size and rarity (Wysocki et al., 2015; Burke et al., 2016a; Burke et al., 2016b; Duvall et al., 2016; Jones, Burke & Duvall, 2014; Orton et al., 2017). In this study, we hypothesized that RGC will provide additional insight into the Group 1 and Group 2 relationships in Poeae, and we will be able to explore RGC as a basis that may explain the division between chloroplast types. Additionally, phylogenies based on RGC data, sequence data, and combined data sets were compared to assess the degree of topological congruence, and determine if plastome-scale RGC are a useful phylogenomic tool either alone or in combination with complete plastome sequences.

We examined Groups 1 and 2 with plastome phylogenomics in two ways that both emphasize deep sampling of molecular characters. (1) We conducted an expanded plastome phylogenomic study with the largest number of plastomes from Poeae analyzed to date (40 species of Poeae in 32 genera of which 16 were newly sequenced) to see if the two subclades of Poeae are consistently inferred when plastome-scale data matrices are analyzed in different ways. (2) We surveyed rare genomic changes (RGC) across all plastomes to determine if specific regions or unusual mutation events in the plastome were disproportionately responsible for the resulting inferences of the Group 1 and Group 2 clades.

## Materials & Methods

### Sampling

The sampling strategy of this study sought to include complete plastomes from all Group 1 and Group 2 genera which (1) had not previously been sequenced, and (2) were situated in positions that were previously under-sampled or contained nonmonophyletic genera.

Specimens were obtained through the USDA, Millennium Seed Bank at the KEW Royal Botanic Gardens (London, United Kingdom), or through collections by colleagues with applicable voucher information (Table 1). This study also expands the Group 2 sampling from the Saarela et al. (2017) study which focused mainly on the Group 1 type species and utilized five selected regions as opposed to the complete plastome analyses as were conducted in this study.

**Table 1:** Sequencing methods & sample procurement information.

**Species**	**Single/ paired end**	**Library prep. method**	**Voucher/PI/MSB/W6**
*Amelichloa brachychaeta*	Single	Nextera XT	PI 197978
*Ammochloa palaestina*	Paired	Nextera	R. Lazaro, s.n. (US)
*Arrhenatherum elatius*	Single	Nextera XT	PI 665562
*Castellia tuberculosa*	Single	Nextera XT	PI 238257
*Catapodium marinum*	Single	Nextera XT	MSB 53132
*Desmazeria sicula*	Paired	Nextera	MSB 17332
*Dichelachne crinita*	Single	Nextera XT	W6 22474
*Festuca ovina*	Paired	Nextera	PI 655206
*Kengyilia melanthera*	Single	Nextera XT	PI 639856
*Koeleria nitidula*	Single	Nextera XT	PI 206688
*Nephelochloa orientalis*	Single	Nextera XT	W6 19223
*Poa* subgen. *Stenopoa*^a^	Paired	Nextera	PI 374046
*Polypogon fugax*	Single	Nextera XT	PI 220619
*Scolochloa festucacea*	Paired	Nextera	Thompson, 866 (ISC)
*Triplachne nitens*	Single	Nextera XT	MSB 26060
*Ventenata macra*	Single	Nextera XT	PI 204431

**Notes.**

T1. Sequencing methods for species included in this study. Procurement information for species sequenced for this study either as seed accessions or as herbarium vouchers. Seed accessions procured from: USDA Plant Introduction (PI), West Regional PI Group (W6), MSB (Kew Millenium Seed Bank).

a *Poa* subgen. *Stenopoa* was originally misidentified as *Festuca pseudovina* (USDA, 2017a). After DNA barcoding of *ETS* and *ITS* nuclear genes, additional plastid genes/IGS (*trnT-trnL-trnF, matK, trnC-rpoB*) were also examined, as well as a morphological assessment; it was determined that this grass is identified as a member of the subgenus *Stenopoa*, however no specific determination could be made as to the exact species.

### DNA extraction

Leaf tissues dried in silica gel were obtained from 16 Poeae species (Table 1) and DNA was extracted by manually homogenizing tissue in liquid nitrogen, followed by using the DNeasy Plant Mini Kit protocol. The Qubit assay (Invitrogen, Thermo Fisher Scientific, Wilmington, DE, USA) was used to quantify total genomic DNAs in the extracts, which were then diluted to 2.5 ng/µl in 20 µl sterile water.

### Illumina Library Prep

The Nextera Prep Kit (Illumina Inc., San Diego, CA, USA; Caruccio, 2011) was used to prepare single-end read DNA libraries for sequencing. Five species (*Festuca ovina, Poa* subgen. *Stenopoa, Scolochloa festucacea, Desmazeria sicula, Ammochloa palaestina*) were also prepared and sequenced from paired-end Nextera libraries because of a lower multiplexing level (Table 1). DNA was purified using the DNA Clean and Concentrator kit (Zymo Research, Irvine, CA, USA), and libraries were prepared using the standard protocol for the respective sample preparation kit. Iowa State University’s Core DNA Facility, Ames, IA, USA, sequenced the libraries on the HiSeq 2500 instrument.

### Plastome Assembly/Annotation

Sequenced plastomes were assembled with exclusively de novo methods, following Wysocki et al. (2014). In processing the next-generation sequencing (NGS) data, DynamicTrim v2.1 of the SolexaQA software suite (Cox, Peterson & Biggs, 2010) was used to perform initial quality trimming on the 99 bp reads using default settings. CutAdapt was used to remove remaining adapter sequences (Martin, 2011). LengthSort v2.1 (Cox, Peterson & Biggs, 2010) was used to remove any sequences shorter than 25 bp in length. CDHit-EST of the CDHit package (Fu et al., 2012) identified and removed redundant sequences; the sequence identity threshold was set at the maximum (parameter: -c 1). This process was automated using a proprietary pipeline script written in Python language (Van Rossum, 1995). The SPAdes v. 3.8.1 software suite (Bankevich et al., 2012) was used for contig assembly. The anchored conserved region extension method was used to scaffold the remaining contigs (Wysocki et al., 2014). Gaps between large contigs were then manually resolved by locating regions of overlap in the quality-trimmed reads until the plastome was completed. Assemblies were verified by mapping the quality-trimmed read pool to the assembled plastome in the Geneious Pro v. 7.1.9 software program (Kearse et al., 2012). Any evidence of apparent errors in the assembly process were identified during verification and manually resolved. Inverted-repeat (IR) boundaries were located by following the methods of Burke, Grennan & Duvall (2012). Each completed plastome assembly was then annotated by initially aligning to closely related Pooideae species, and annotations were transferred from the reference to the newly assembled plastome (Wysocki et al., 2014). Protein coding sequences were adjusted when necessary to correctly position coding sequence boundaries and preserve reading frames.

### Mutation analyses

A matrix of 16 newly completed (Table 2) and 26 previously published Poeae plastomes (Table 3) were aligned in Geneious Pro using the MAFFT v6.814b plug-in (Katoh & Standley, 2013), and all column gaps that were introduced by the alignment process were removed before analysis, but after RGCs had been characterized. The second copy of the inverted repeat (IRa) was also removed to eliminate redundancies. This stripped matrix was analyzed together with outgroups (Triticeae: *Kengyilia melanthera* and Stipeae: *Amelichloa brachychaeta*) to determine initial group membership. This complete analysis of all taxa was done to reduce any biases due to sampling, taxonomy, or outgroup selection. Outgroup species were chosen based on the results of previous studies (Soreng et al., 2015; Soreng et al., 2017; Saarela et al., 2015).

**Table 2:** Plastome characteristics for sequenced species.

Species	GenBank accession	Plastome length	IR length	LSC	SSC	%AT
*Amelichloa brachychaeta*	MH569074	139,946	21,571	83,936	12,868	62.0
*Ammochloa palaestina*	MH569075	135,887	21,543	80,156	12,646	61.7
*Arrhenatherum elatius*	MH569076	136,233	21,633	80,399	12,569	61.6
*Castellia tuberculosa*	MH569077	133,798	21,241	78,820	12,497	61.6
*Catapodium marinum*	MH569078	134,366	21,565	78,567	12,670	61.8
*Desmazeria sicula*	MH569079	133,982	21,565	78,204	12,649	61.7
*Dichelachne crinita*	MH569080	136,278	21,663	80,315	12,638	61.4
*Festuca ovina*	MH569081	133,569	21,237	78,698	12,398	61.7
*Kengyilia melanthera*	MH569082	135,642	21,562	79,773	12,747	61.6
*Koeleria nitidula*	MH569083	136,085	21,635	80,251	12,564	61.4
*Nephelochloa orientalis*	MH569084	135,468	21,504	79,788	12,673	61.8
*Poa* subgen. *Stenopoa*^a^	MH569085	135,362	21,544	79,626	12,649	61.6
*Polypogon fugax*	MH569086	136,639	21,670	80,540	12,759	61.2
*Scolochloa festucacea*	MH569087	134,087	19,402	82,569	12,715	61.5
*Triplachne nitens*	MH569088	134,457	19,563	82,737	12,594	61.5
*Ventenata macra*	MH569089	135,784	21,512	80,067	12,694	61.7

**Notes.**

T2. Information defining species sequenced for this study; including: GB accession number and relevant plastome statistics.

a Refer to Table 1 for additional information on the identification of *Poa* subgen. *Stenopoa*.

**Table 3:** Previously published plastomes.

Species	GenBank Accession	Reference
*Agrostis gigantea*	MF460976	Saarela et al. (2018)
*Agrostis stolonifera*	NC_008591	Saski et al. (2007)
*Alopecurus arundinaceus*	NC_037163	Saarela et al. (2018)
*Anthoxanthum odoratum*	NC_027467	Saarela et al. (2015)
*Avena sativa*	NC_027468	Saarela et al. (2015)
*Briza maxima*	KM974736	Saarela et al. (2015)
*Calamagrostis breviligulata*	NC_027465	Saarela et al. (2015)
*Catapodium rigidum*	NC_036711	Saarela et al. (2018)
*Dactylis glomerata*	NC_027473	Saarela et al. (2015)
*Deschampsia antarctica*	NC_023533	Lee et al. (2014)
*Festuca altissima*	NC_019648	Hand et al. (2013)
*Festuca arundinacea*	KM974751	Saarela et al. (2015)
*Festuca ovina*	NC_019649	Hand et al. (2013)
*Festuca pratensis*	NC_019650	Hand et al. (2013)
*Gastridium ventricosum*	NC_036686	Saarela et al. (2018)
*Helictochloa hookeri*	NC_027469	Saarela et al. (2015)
*Hierochloe odorata*	KM974740	Saarela et al. (2015)
*Holcus lanatus*	NC_036689	Saarela et al. (2018)
*Lamarckia aurea*	NC_037168	Saarela et al. (2018)
*Lolium multiflorum*	NC_019651	Hand et al. (2013)
*Lolium perenne*	NC_009950	Diekmann et al. (2009)
*Phalaris arundinacea*	NC_027481	Saarela et al. (2015)
*Poa palustris*	NC_027484	Saarela et al. (2015)
*Puccinellia nuttalliana*	NC_027485	Saarela et al. (2015)
*Torreyochloa pallida*	NC_027486	Saarela et al. (2015)
*Trisetum cernuum*	NC_027487	Saarela et al. (2015)

**Notes.**

T3. GenBank accession numbers and reference studies for previously published species included in this study.

RGCs were identified manually, following the methods of Leseberg & Duvall (2009) and Orton et al. (2017), and evaluated to determine if the event was non-ambiguous and whether or not homology could be reliably assessed across the dataset. Ambiguous events were defined as an event that could not be reliably inferred as either ancestral or derived based on sequence evidence across multiple species exhibiting a specific event. This ambiguity is likely an artifact of multiple mutations occurring in the same region of sequence, obscuring any ability to infer a mutational mechanism or clearly identify a RGC event. RGCs identified as ambiguous were subjected to stringent culling procedures to ensure that no biases (either through the alignment, or manual recognition) existed in determination of ambiguous events. The RGC were then examined to determine if they were attributed to a specific cause such as slipped-strand mispairing (SSM) or intrastrand deletion (ISD) events.

SSM events occur when there are tandem repeats in sequences allowing indels to arise. ISD are characterized by a deletion in one sequence, which when aligned with similar sequences lacking the deletion, indicate direct dispersed repeats exactly flanking the deletion (Graur, Sater & Cooper, 2016). Recombination events may leave little to no sequence evidence, however they are a likely mechanism resulting in RGC given their size and low occurrence (Graur, Sater & Cooper, 2016).

A binary matrix was then produced to indicate the ancestral state (0) and the derived character state (1) for each RGC. The condition of the ancestral state was assumed to be that of the outgroup. Binary matrices did not contain sufficient informative characters to act as a stand-alone data set. However, these binary matrices were combined with the sequence data sets for Group 1 only, Group 2 only, and the Group 1 and 2 analyses (combined data sets).

In total, 12 data sets (6 maximum likelihood; 6 Bayesian inference) were constructed for Group 1, Group 2, and Group 1 & 2, which included the stripped sequence only alignment, and a combined data set of stripped sequence and binary data for maximum likelihood and Bayesian inference analyses.

### Phylogenomic analyses

A jModelTest v2.1.3 (Darriba et al., 2012) analysis was performed using the Group 1, Group 2, and Group 1 & 2 aligned and stripped sequence data sets to determine the appropriate model under the Akaike Information Criterion (Akaike, 1974; Posada, 2008). The GTR + I + G model was within the 100% confidence interval and thus, was selected.

A total of 12 analyses were performed using maximum likelihood (ML; six trees produced), and Bayesian Inference (BI; six trees produced). There were two separate matrices for Group 1, Group 2, and Group 1 & 2: (1) plastome nucleotide sequences only, and (2) plastome sequences concatenated with the RGC data set. Trees were visualized using the TreeGraph2 program (Stöver & Müller, 2010). With a maximum of 38 binary characters, the RGC dataset was not analyzed separately as there were not enough informative characters to produce resolved phylogenies.

ML analyses were performed with the RAxML-HPC2 v8.1.11 program on XSEDE (Stamatakis, 2014) through the CIPRES science gateway (Miller, Pfeiffer & Schwartz, 2010). A ML tree was produced for each matrix and a consensus bootstrap tree was constructed from 1,000 replicates using the Consense tool from the Phylip v3.66 software suite (Felsenstein, 2005) available on the CIPRES science gateway. The combined sequence and binary analyses were partitioned into the sequence and binary matrices, and a multistate CAT model was selected for both nucleotide (GTR) and binary (BIN) data to maintain a fixed number of substitution rate categories.

BI analyses with two independent chains were performed for each data partition on XSEDE via the CIPRES science gateway using the MrBayes v3.2.6 program (Ronquist et al., 2012). The MCMC analyses were run at 10 million generations with each chain sampled after 1000 generations. The data type was set to “restriction” for the binary partition of the data when it was included with sequence data (Ronquist & Huelsenbeck, 2003). The R-package “RWTY” v.1.0.2 (Warren, Geneva & Lanfear, 2017) was used to assess convergence.

## Results

For this study, 2,167,574 bases were newly sequenced in 16 Poeae plastomes, spanning 11 subtribes, with lengths ranging from 133,569 to 139,946 bp (Tables 1 and 2). All plastomes were deposited in the NCBI GenBank database (accession numbers MH569074 –MH569089). One plastome (*Arrhenatherum elatius*) contained two regions that could not be assembled, which were estimated to be 10 and 36 bp by comparison with *Avena sativa* (KM974733) as reference, falling in the intergenic regions on either side of *ndhF*. Both gaps occurred in regions with greater than 73% AT richness. Illumina library preparation kits and/or sequencing have been found to have a bias against AT rich regions (Burke et al., 2016a; Burke et al., 2016b).

Overall, 156 RGC events were determined to meet our criteria to be classified as RGC. After assessing homology, 38 RGC were determined to be alignable across all taxa and were subsequently analyzed. Two RGC were flanked by dispersed repeats in reference sequences suggesting ISD. The remaining RGC were likely derived through recombination events, as there is no sequence evidence available to evaluate the mutation mechanism that resulted in the event (Graur, Sater & Cooper, 2016). Non-ambiguous RGC ranged in length from 50 to 543 bp with an average length of 127 bp.

### Plastome phylogenomic analysis

In plastome phylogenomic analyses, BI topologies were congruent to ML topologies and will not be considered separately; all reported support values are ML bootstrap (BS) values (see (Figs. S1–S3; Table S1) for BI analyses and corresponding tree topologies).

#### Group 1 & 2

**Figure 1:**
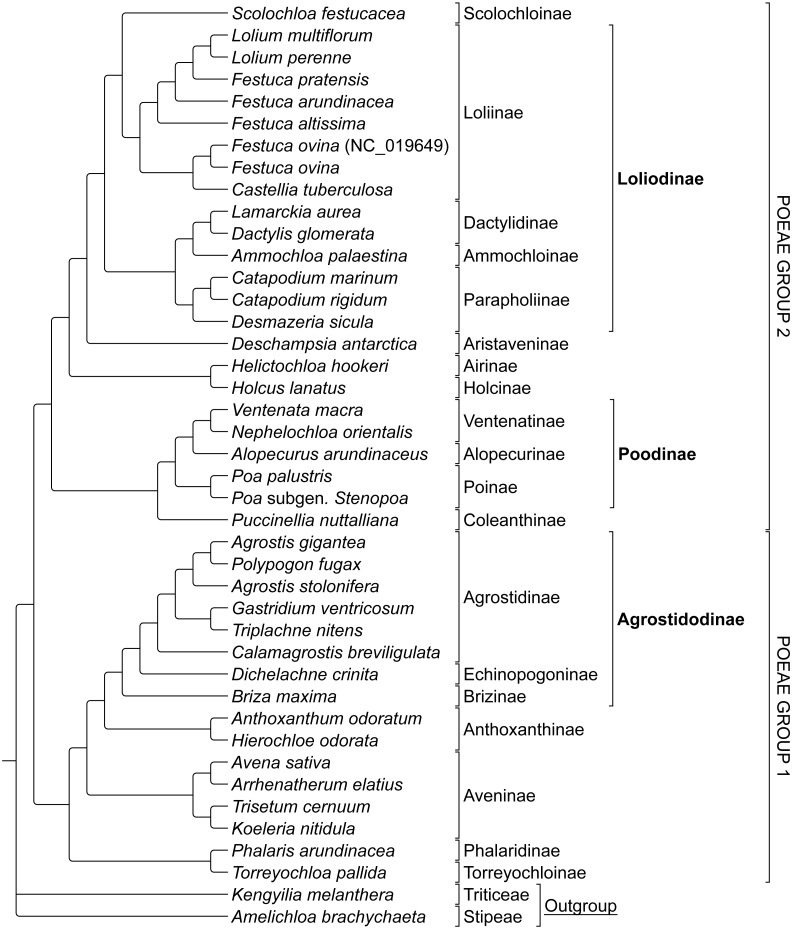
Phylogeny of Poeae group 1 and 2 species included in this study. ML cladogram showing relationships between both Group 1 and Group 2 taxa. Bracketed clades correspond to taxa sensu Soreng et al. (2017). GenBank accession number indicates the previously published species of *F. ovina.* Supersubtribe designations are shown in bolded text. Subtribe designations are shown in standard text.

The combined alignment of species in Groups 1 and 2 was analyzed first to prevent bias based on the predetermined memberships of Group 1 and 2 plastome species retrieved in previously published research. The stripped alignment of 42 Poeae species was 97,820 bp in length. The ML tree produced a topology congruent to those of previous studies, insofar as comparisons were possible given somewhat different sampling. Presumed Group 1 taxa were sister to those in Group 2. *Amelichloa brachychaeta* and *Kengyilia melanthera* were outgroups for Group 1 and Group 2 (Fig. 1).

**Figure 2:**
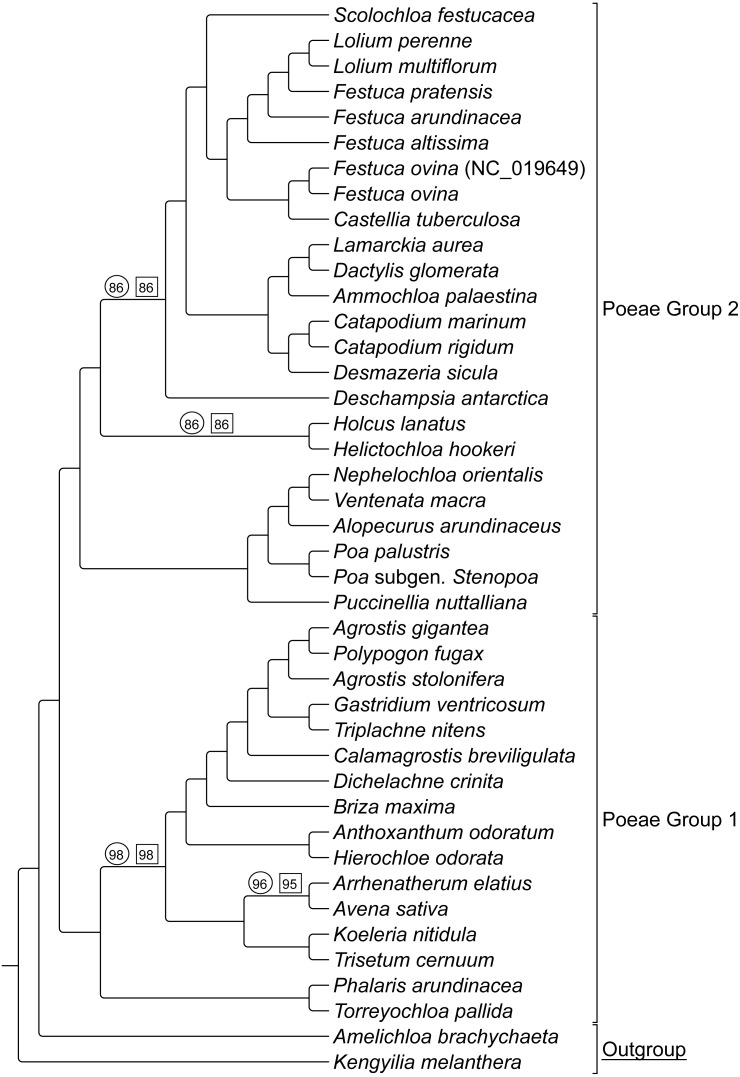
ML sequence and RGC BS consensus for group 1 and 2 taxa. ML consensus cladogram of sequence and rare genomic change (RGC) data for Group 1 and Group 2 taxa with bootstrap support (BS) values noted. Nodes without values are at maximum support. Support values within circles correspond to sequence only analyses. Support values within squares correspond to sequence + RGC analyses. GenBank accession number indicates the previously published species of *F. ovina. A. brachychaeta* and *K. melanthera* serve as outgroup comparisons.

ML bootstrap consensus tree results had support values of 100% for all nodes, except: (1) the (*Holcus + Helictochloa*) clade [86%], (2) the monophyly of *Deschampsia* plus (Loliodinae + *Scolochloa*) [86%], (3) the monophyly of all included Group 1 taxa except (*Phalaris + Torreyochloa*) [98%] and (4) the position of *Avena sativa* as sister to *Arrhenatherum elatius* [96%] (Fig. 2). Novel Group 1 relationships from plastome data are: *Agrostis stolonifera* is sister to (*Agrostis gigantea* + *Polypogon fugax*) so that *Agrostis* is nonmonophyletic. The (*Gastridium ventricosum* + *Triplachne nitens*) clade is maximally supported as sister to the *Agrostis*-*Polypogon* complex, which is in turn sister to *Calamagrostis breviligulata*. Group 2 relationships of note are: *Castellia tuberculosa* is embedded within the (*Festuca* + *Lolium*) clade. *Ammochloa palaestina* is sister to Dactylidinae (*Dactylis glomerata* + *Lamarckia aurea*) (Fig. 2).

An additional combined matrix including both sequence and RGC for Groups 1 and 2 resulted in a ML topology fully congruent with the sequence only data set. Bootstrap support values were 100% for all nodes except the same four nodes just described above with support values of 86%, 86%, 98%, and 95% respectively (Fig. 2).

Group 1 & 2 RGC events were analyzed to determine if any events were shared between both groups, and it was determined that four events were not restricted to one group or the other indicating homoplasy relative to these two groups (see Table S2- Taxonomic Markers, for more information).

#### Group 1

**Figure 3:**
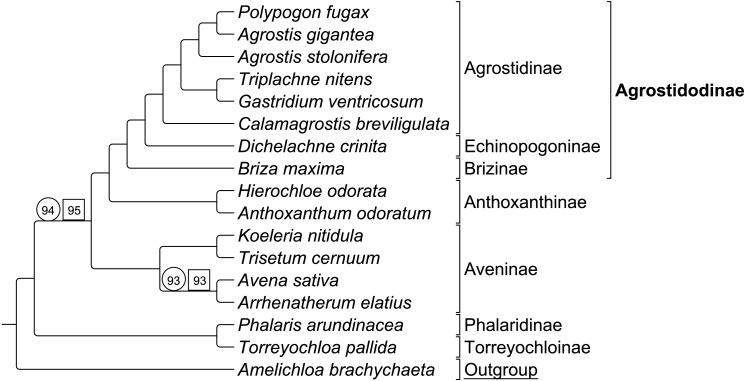
ML BS consensus tree of Group 1 species. ML BS tree of Group 1 species inferred from aligned sequences. The support values of two nodes, which are less than maximum, are indicated. Support values within circles correspond to sequence only analyses. Support values within squares correspond to sequence + rare genomic change (RGC) analyses. *A. brachychaeta* serves as the outgroup comparison. Supersubtribe designations are shown in bolded text. Subtribe designations are shown in standard text. Bracketed clades correspond to taxa sensu Soreng et al. (2017).

Group 1 sequence only taxa set analyzed separately exhibited topological congruence to that of previous studies (Soreng et al., 2015; Soreng et al., 2017; Saarela et al., 2015) (Fig. 3). Bootstrap support values were 100% for all nodes with two exceptions. The node uniting the Aveninae clade and the (Anthoxanthinae + Agrostidodinae) clade was supported at 94%, and the node uniting *Avena sativa* and *Arrhenatherum elatius* as sister taxa was supported at 93% (Fig. 3).

Group 1 combined data set included both sequence and RGC matrix for group 1 only. The ML tree resulted in a topology congruent with the sequence only data set and with phylogenies of previous studies. Bootstrap support values were 100% with the two exceptions noted above, and support was recorded at 95%, and 93%, respectively (Fig. 3).

Six synapomorphic characters were identified in the Group 1 dataset (Table S2-Taxonomic Markers). As with the combined RGC dataset, the number of informative characters was too few to provide useful stand-alone phylogenetic information, and thus a separate analysis of RGC data alone was not conducted.

#### Group 2

**Figure 4:**
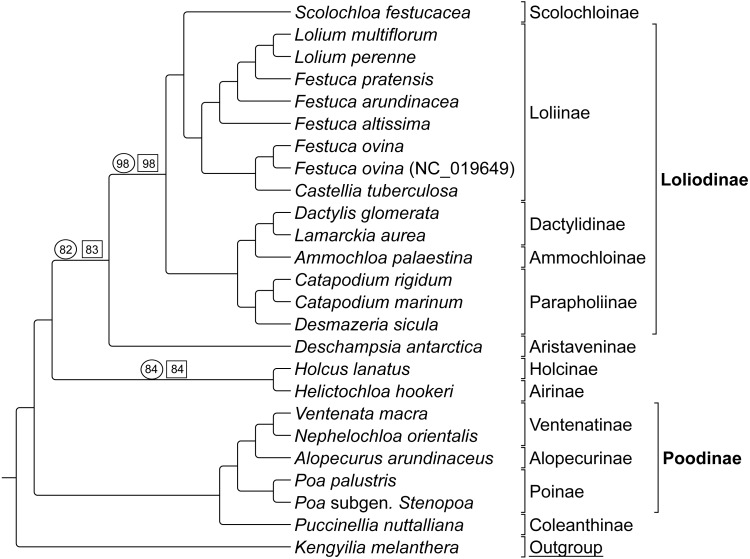
ML BS tree of Group 2 species inferred from aligned sequences. The support values of three nodes, which are less than maximum, are indicated. Support values within circles correspond to sequence only analyses. Support values within squares correspond to sequence + rare genomic change (RGC) analyses. GenBank accession number indicates the previously published species of *F. ovina. K. melanthera* serves as the outgroup comparison. Supersubtribe designations are shown in bolded text. Subtribe designations are shown in standard text. Bracketed clades correspond to taxa sensu Soreng et al. (2017).

Group 2 sequence analyses also showed congruent topologies (Fig. 4) compared to the combined Group 1 and 2 sequence only analysis performed first. Bootstrap support values for Group 2 taxa were 100% with the following exceptions: (1) the node uniting *Deschampsia* with (*Scolochloa* + Loliodinae) was supported at 98%, (2) the node supporting *Holcus* and *Helictochloa* as sister to *Deschampsia* + (*Scolochloa* + Loliodinae) at 82%, and (3) the node supporting *Holcus* and *Helictochloa* at 84% (Fig. 4).

As with Group 1 combined data, Group 2 taxa analyzed with combined sequence and RGC data resulted in a topology congruent with the Group 2 sequence only analysis (Fig. 4). Bootstrap support values for Group 2 combined data were supported at 100% with three exceptions. The node uniting *Deschampsia* with (*Scolochloa* + Loliodinae) resulted in 98% support, the node defining (*Holcus* + *Helcitochloa*) as sister to *Deschampsia* + (*Scolochloa* + Loliodinae) was supported at 83%, while the node supporting *Holcus* and *Helictochloa* was 84% (Fig. 4).

Group 2 contained 27 synapomorphic RGC events (Table S2-Taxonomic Markers) for species represented in the Loliodinae, Ventenatinae, Alopecurinae, and Poinae (sensu Soreng et al., 2017).

Analyses conducted for this study produced near identical support for taxa relationships across ML and BI methods. Additional statistics (-log likelihood, standard deviation of bootstrap (ML), standard deviation of split frequencies (BI)) and mean bootstrap (ML)/posterior probability (BI) values for plastome phylogenomic trees based on analyses (ML, BI) and dataset partitions (sequence, and combined sequence + binary data) are listed in Table S1.

### Identification of RGC or unique genome features

RGC in the combined Group 1 & 2 data sets were not always identical to those in the separate alignments of Group 1 and Group 2 (Table S2). This was due to the divergence between Group 1 and Group 2 taxa altering the sequence alignment and masking some individual RGC events. In characterizing the RGC data from the combined matrix, there were four RGCs identified as homoplasious between Groups 1 and 2, after ambiguous RGC were discarded. There were six RGC in the alignment of Group 1 found to be synapomorphic for Group 1 clades, and 27 RGC found to be synapomorphic for clades in Group 2.

The RGC data were also examined to determine whether mutations were the result of ISD or SSM. It was determined that two of the events originated in sequence through ISD, but no event was the result of SSM. Both *Polypogon fugax* and *Briza maxima* exhibited unique ISD events. The ISD event occurring in *P. fugax* was between CDS *psbE* and *petL* and is 63 bp in length (Table 4).

**Table 4:** Sequence evidence for Intrastrand Deletion event in Group 1 taxa (in sequence alignment, bases: 69,883–69,962).

*Amelichloa brachychaeta*	TAATCCAAAATAGAAATAAC---CTTTTTTTTTCTAATTCAATT----CTTTATTTATCTCTTATTCC-----AAAATTC
*Triplachne nitens*	TAA**TCCAAAATTC**AATT**GTTTA**-TTTTTTTTTGCAAATTCAATTGTTTT**GTTTA**TTTATCTCTTATTCC----AAAATTC
*Gastridium ventricosum*	TAA**TCCAAAATTC**AATT**GTTTA**-TTTTTTTTTGCAAATTCAATTGTTTT**GTTTA**TTTATCTCTTATTCC----AAAATTC
*Dichelachne crinita*	TAATCCAAAATAGAA---AGCATTTTTTTTTTTCAAATTCAATT-----GTTTATTTATCTCTTATTCC----AAAATTC
*Calamagrostis breviligulata*	TAA**TCCAAAATTC**AATTGTTTATTTTTTTTTTTCAAATTCAATT-----GTTTATTTATCTCTTAT**TCC----AAAATTC**
*Polypogon fugax* (ISD)	TAA---------------------------------------------------------------**TCC----AAAATTC**
*Agrostis gigantea*	TAA**TCCAAAATTC**AATT**-----**---------------------------**GTTTA**GTTATCTCTTATTCC----AAAATTC
*Agrostis stolonifera*	TAA**TCCAAAATTC**AATT**-----**---------------------------**GTTTA**TTTATCTCTTATTCC----AAAATTC
*Hierochloe odorata*	TAATCCAAAATAGAAAGCATT--TTTTTTTTTTCAAATTCAATT-----GTTTATTTATTTCTTATTCC----AAAATTC
*Torreyochloa pallida*	-------------------------------------------------GTTTATTTATCTCTTATTCC----AAAATTC
*Phalaris arundinacea*	TAATCCAAAATAGAAAGCA----TTTTTTTTTTCAAATGCAATT-----GTTTATTTATCTCTTATTCCAAAAAAAATTC
*Koeleria nitidula*	TAATCCAAAATAGAAAACA-T--TTTTTTTTTTCAAATTCAATT-----GTTTACTTATCTCTTATTCC----AAAATTC
*Trisetum cernuum*	TAATCCAAAATAGAAAACA-T--TTTTTTTTTTCAAATTCAATT-----GTTTATTTATCTCTTATTCC----AAAATTC
*Avena sativa*	TAATCCAAAATACAAAACATT--TTTTTTTTTTCAAATTCAATT-----GTTTATTTATCTCTTATTCC----AAAATTC
*Arrhenatherum elatius*	GAATCCAAAATAGAAAACA-------TTTTTTTTTAATTCAATT-----GTTTATTTATCTCTTATTCC----AAAATTC
*Anthoxanthum odoratum*	----------------------------TTTTTCAAATTCAATT-----GTTTATTTATCTCTTATTCC----AAAATTC
*Briza maxima*	-------------------------------------------------GTTTATTTATTTATTATTCC----AAAATTC

**Notes.**

T4. Alignment evidence showing the ISD event in *Polypogon fugax*. Nucleotides of importance are bolded or underlined.

**Table 5:** Sequence evidence of 78 bp ISD in Festuca ovina versus previously published *Festuca ovina* (NC_019649).

*Festuca ovina* (NC_019649)	AGG------------------------------------------------------------------------------**AAAAAGAAA**TTC
*Festuca ovina*	AGG**AAAAAGAAA**GAA-----AAGATGGATTGGGTTGAACCTCAGAGTCATTAAAAATAGGGTA----AATTCTATTTTGGAAAAAAGAAATTC
*Festuca altissima*	AGG**AAAAAGAAA**GAA-----AAGATGGATTGGGTTGAACCTCAGAGTCATGAAAAATTTGGTA----AATTCTATTTTGGAAAAAAGAAATTC
*Festuca arundinacea*	AGG**AAAAAGAAA**TAA-----AAGATGGATTGGGTTGAACCTCAGAGTCATGAAAAATTTGGTA----AATTATATTTTGGAAAAAAGAAATTC
*Festuca pratensis*	AGG**AAAAAGAAA**TAA-----AAGATGGATTGGGTTGAACCTCAGAGTCATGAAAAATTTGGTA----AATTATATTTTGGAAAAAAGAAATTC

**Notes.**

T5. Alignment evidence of the 78 bp ISD event in Festuca ovina (NC_019649) not seen in *F. ovina* sequenced for this study, indicating a potential for these events to occur independently within a species.

In identifying unique genome features, this study utilizes data from 40 Poeae taxa in 34 genera including one instance of two accessions of the same species, *Festuca ovina,* which can be compared. The previously published *F. ovina* contains a 78 base deletion, located in the IGS between *psaJ* and *rpl* 33 CDS, which is not seen in the *F. ovina* sequenced for this study (Table 5). This deletion also shows the characteristic dispersed repeats associated with ISD events. The two *F. ovina* species have 99.3% sequence identity (132,816 identical sites of 133,709 total sites) indicating these are conspecific. Furthermore, the 78 base indel shows an average read depth of 32.1 in the *F. ovina* species assembled for this study, providing strong support for inserted sequence in this region not seen in the previously published specimen. The presence of this ISD event in the previously published *F. ovina* (NC_019649) and its absence in a newly published individual of the same taxon indicates a potential for these events to occur independently within a species, as evidenced by our two *F. ovina* representatives.

## Discussion

During the course of this study, it was determined that RGC are potentially useful as taxonomic markers to identify clades. The relationships of Group 1 and Group 2 species, as described in previous research (Saarela et al., 2015; Soreng et al., 2015; Soreng et al., 2017) with fewer species and/or fewer loci, have been confirmed, but with robust support and updates due to the increase in sampled taxa in this study (e.g., *Castellia* and *Ammochloa*) as compared to previous studies, as well as deep sampling of the plastome. In utilizing the complete plastome data, more genetic information is available and provides a more accurate picture of phylogenomic relationships within Poeae. Support values for sequence only data, and sequence data combined with binary RGC data provide robust support for relationships of Group 1 and Group 2 taxa, and resolution of previously unsupported or unresolved relationships within groups.

### Phylogenomic analyses

The phylogenomic analyses conducted in this study, provided insight into the relationships in Pooideae. While our analysis is consistent with the circumscription of Agrostidinae sensu Soreng et al. (2017), *Calamagrostis, Briza, and Dichelachne* are united with this clade with maximum support, despite the absence of plastome data from the Calothecinae subtribe. This is an increase compared to moderate support for the same clade in Saarela et al. (2017). Our results are also consistent with the broader circumscription of the subtribe by Kellogg (2015). Within the subtribe the *Gastridium* + *Triplachne* clade is maximally supported as sister to the *Agrostis*-*Polypogon* complex, which is in turn sister to a species of *Calamagrostis*. Additional sampling of complete plastomes among the remaining genera of Agrostidinae will further inform the efforts to classify this group.

Of particular note, *Agrostis* is nonmonophyletic in our plastome trees. This study resolved the Group 1 taxon *Polypogon fugax* as sister to *Agrostis gigantea,* with *Agrostis stolonifera* sister to those in turn, all with maximum support. To verify the identity of our unvouchered seed source of *Polypogon*, we compared our plastome sequence to GenBank submissions of 13 plastid barcode markers (accession numbers: EU639581, HQ599932, KF796891, KF797152, KF797264, KF797017, KP135424, KP135426, KX372479, MF064763, MF065677, MF073532 and MF785855) sequenced from other tissue sources of *P*. *fugax* using BLASTn. In nine cases, nucleotide identity was 100% between our plastome and the barcodes of *P*. *fugax* and in the remainder it was 99%. In all cases, comparisons of our sequence with the *Agrostis* plastid sequences available through NCBI (Altschul et al., 1990) were <100% (mean identities for both *Agrostis* species: 97%) confirming the identifications of *Polypogon* and *Agrostis* in this study. This confidently inferred relationship based on new plastome data should be explored further and possibly used as the basis for reclassification.

Suspected hybridizations in the *Lolium*-*Festuca* complex in Group 2 make the discernment of exact relationships difficult. Here, *Festuca* species do not form a monophyletic group, which is fully consistent with the circumscription of taxa in Soreng et al. (2017). Instead, two accessions of *Festuca ovina* are sister to each other and are united with *Castellia tuberculosa* in a separate clade from the remaining *Festuca* + *Lolium* species. Note that in a three-locus plastid study, Bouchenak-Khelladi et al. (2009) strongly supported *C. tuberculosa* as sister to their (*Vulpia* + *Festuca*) clade. However, the earlier ITS, *trn* L-F study conducted by Catalán et al. (2004) found conflicting positions among the ITS and *trn* L-F analyses, in which the plastid loci mirrored the position of *C. tuberculosa* returned in our study in relation to *Festuca* (Fig. 1). *Festuca pratensis* (*Lolium pratense* sensu Saarela et al., 2018) is sister to the Lolium clade, while *Festuca arundinacea* (*Lolium arundinaceum* sensu Saarela et al., 2018) is sister to the latter clade, and finally, *Festuca altissima* (*Drymochloa sylvatica* sensu Saarela et al., 2018) is situated on a long branch and sister to the aforementioned groupings in the Loliinae.

An additional point of interest in the results of this study is the unique placement of the Group 2 taxon *Scolochloa festucacea.* Here, *S. festucacea* is embedded within the Loliodinae, and sister to the Loliinae with maximum support (Figs. 2 and 4), contrary to the circumscription of Soreng et al. (2017) which excludes Scolochloinae from the Loliodinae. To confirm the identity of our vouchered specimen, we compared our plastome sequence to four plastid barcode markers produced in different studies from other sources of *S. festucacea* available through NCBI GenBank (AM234600, KJ913040, KM524033, and KM524103). In all comparisons, nucleotide identity was 99.4% or greater, with a maximum identity of 99.9% and 1124/1125 identical sites (KM524033; *rpo*B-*trn* C), thus confirming the identity of *S. festucacea* in this study.

*Ammochloa* has not been previously included in plastome phylogenomic studies. Here we find a strongly supported position for *Ammochloa* sister to Dactylidinae. This confirms the weakly supported topology inferred for these same taxa in Quintanar, Castroviejo & Catalán (2007), although in their study this clade is in an unresolved position whereas our results place it sister to (*Catapodium* + *Desmazeria*). Results of Saarela et al. (2010) based on ITS data, moderately supported the position of *Ammochloa* in a paraphyletic grade of Airine and other Group 2 taxa. The inclusion of representatives of *Ammochloa* and *Castellia* here, which have been little studied, indicate affinities in Loliodinae.

### RGC data set

**Figure 5:**
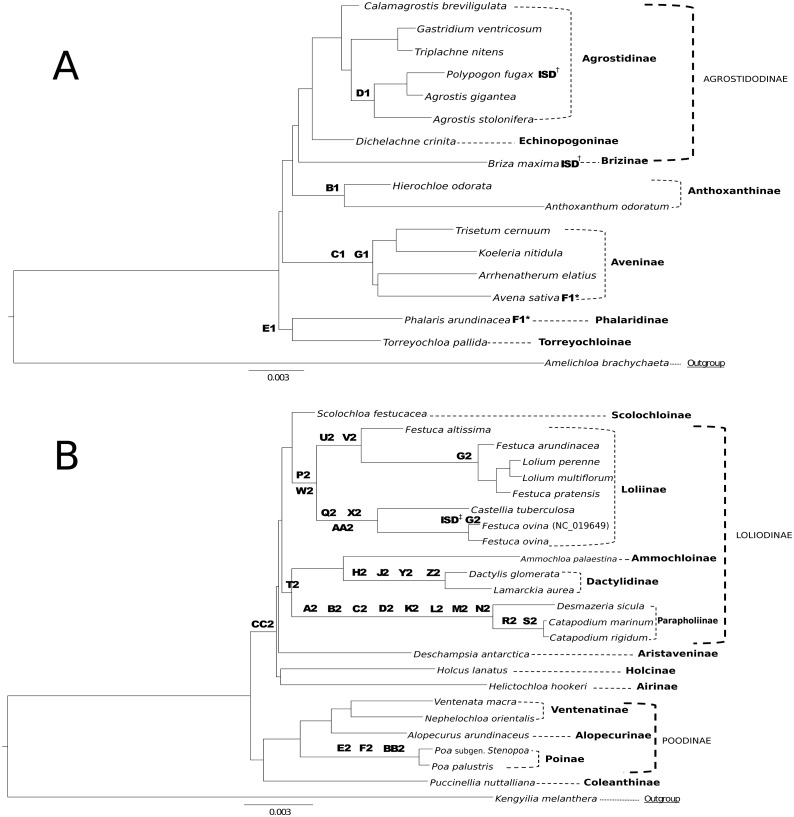
Taxonomic markers identified for Group 1 & Group 2 taxa. (A) Taxonomic markers including the unique intrastrand deletion (ISD) event in *P. fugax* and *B. maxima*, are superimposed onto a ML phylogram for Group 1. Scale bar length is 0.003. Letter-number codes correspond to “Taxonomic Markers” found in Table S2 for Group 1 taxa. Subtribes are designated by dashed-line or bracket and bolded text; supersubtribes are designated by bold dashed-line/bracket and capitalized text. Bracketed clades correspond to taxa sensu Soreng et al. (2017). One rare genomic change (RGC) (F1) appears as homoplasious in both *Phalaris arundinacea* and *Avena sativa*. † Indicates a unique ISD event in the designated taxa. These ISD events are synapomorphic. (B) Taxonomic markers including the ISD event in *F. ovina,* are superimposed onto a ML phylogram for Group 2. Scale bar length is 0.003. GenBank accession number indicates the previously published species of *F. ovina.* Letter-number codes correspond to “Taxonomic Markers” found in Table S1 for Group 2 taxa. Subtribes are designated by dashed-line or bracket and bolded text; supersubtribes are designated by bold dashed-line/bracket and capitalized text. Bracketed clades correspond to taxa sensu Soreng et al. (2017). ‡ Indicates the unique ISD event seen in the previously published *F. ovina* (NC_019649); see Table 5 for sequence evidence of this ISD event.

The RGC analysis identified 156 non-ambiguous individual events, of which 33 were determined to be clade defining markers within Group 1 or Group 2 (Table S1 Taxonomic Markers). We propose use of these RGC as taxonomic markers (Figs. 5A & 5B), because they show little homoplasy. Two of the RGC, F1 and G2, show convergence/parallelism on our trees (Figs. 5A & 5B). Of the total RGC, 87% were synapomorphic to subgroups of either Group 1 or Group 2 in a combined alignment of all 42 plastomes. Only four RGC were homoplasious characters identified across the Group 1 and Group 2 alignment. Complete RGC sequences and letter-number codes for these events can be found in (Table S2 Taxonomic Markers).

Analysis of RGC improves our understanding of molecular evolution in the plastome. RGC were analyzed and characterized to determine if they had arisen through identifiable mutation events such as ISD or SSM. Two RGC were interpreted to be the result of ISD based on evidence seen in the aligned sequence. However, these occurrences of ISD are relatively uncommon. Given the estimated divergence of Pooideae at 52 Mya (Burke et al., 2016a; Burke et al., 2016b), this may have provided ample time to eliminate dispersed repeats with subsequent mutations so that ISD events can no longer be distinguished from nonreciprocal recombinations.

RGC events shared by closely related species or clades can be useful and inferential for phylogenomic relationships. This is demonstrated in Figs. 5A & 5B, where RGC are indicated on the corresponding nodes of the tree. Group 1 (Fig. 5A) contained a total of six clade defining RGC events and Group 2 (Fig. 5B) contained 27 clade defining RGC events; these events variously support tribal, multi-tribal, and within-tribal relationships in Group 1: Aveninae, Agrostidinae, Anthoxanthinae, (Phalaridinae + Torreyochloinae); and Group 2: Loliinae, (Ammochloinae + Parapholiinae + Dactylidinae), Parapholiinae, Dactylidinae, Poinae, and Coleanthinae.

Group 2 taxa displayed a unique trend in the high occurrence of taxonomic markers situated on long branches (Fig. 5B). This is particularly noticeable between *Desmazeria sicula* and the *Catapodium* species, where there are eight RGC taxonomic markers. Additionally, taxonomic markers: CC2, P2/W2, and T2 result in divided clades. The taxonomic marker CC2 is synapomorphic for Group 2 subtribes Loliodinae + (Airinae, Holcinae, and Aristaveninae) and excludes the (Poodinae + Coleanthinae). Loliinae is separated from the remainder of Loliodinae subtribes by a pair of mutations: P2 and W2; and the final T2 mutation is synapomorphic for (Ammochloinae + Dactylidinae + Parapholiinae) and excludes the Loliinae.

In general, the inference of monophyletic Group 1 and Group 2 clades is not constrained to a few specific regions of the plastome, as these RGC events are found distributed across the plastome. With the deep sampling of the plastome conducted in this study, there is not a specific attributable RGC or small set of these that are responsible for the reciprocal monophyly of Group 1 and Group 2 indicating that perhaps the division between these two plastid groups is an ancient event, long since masked by the subsequent molecular evolution of the plastomes of these groups.

## Conclusion

This study has exhaustively characterized complete plastomes and RGC in Poeae Groups 1 and 2, based on all available genetic information in the plastid chromosome. Most of the previous studies examining the relationships between Group 1 and Group 2 Poeae species utilized a few genes or loci, while this study has considered all available genetic information of the plastome. Because of this deep sampling of the plastome, the results of this study are strongly supported and infer some previously unidentified relationships, while also confirming many of the previously determined relationships. RGC are an additional and useful set of data to consider when examining relationships between taxa, especially as taxon sampling increases. Additionally, RGC provide further utility as training data for algorithms created to identify these mutations and mutational mechanisms. Overall, through the use of complete plastomes, this study demonstrated robust support for the relationships of Poeae Group 1 and 2, as well as explored the use of RGC as promising broad scope, clade defining characteristics for taxa within the Poeae. Future directions might include investigations of substitution rates in regions prone to RGC and a larger and deeper sampling across the entire Pooideae to determine relationships across the group, and also by including nuclear data analyzed with coalescent methods. In particular, the unique placements of *Scolochloa festucacea* within the Loliodinae, and *Polypogon fugax* as paraphyletic with *Agrostis* bears further examination. Note that while biparentally inherited nrDNA is a theoretically better source for inferring species phylogenies, in the case of the highly reticulate Poeae the use of nuclear loci complicates phylogenetic reconstruction or can cause inference of inaccurate species phylogenies due to incorrect interpretation of recombination events or extensive polyploidizations (Álvarez & Wendel, 2003; Saarela et al., 2017). However, genome-scale nuclear analyses are more likely to overwhelm errors of interpretation with phylogenetic signal and lead to a better understanding of the complex evolution in this highly reticulate group.

##  Supplemental Information

10.7717/peerj.6959/supp-1 Supplemental Information 1 BI analyses BI trees for Group 1&2, Group 1, and Group 2 taxa.

10.7717/peerj.6959/supp-2 Table S1 Additional data describing ML and BI analyses conducted in this study SI.T.1 Data describing ML and BI analyses and resulting phylogenomic trees. G1, Group 1; G2, Group 2; RGC, Rare Genomic Changes.

10.7717/peerj.6959/supp-3 Table S2 RGC dataset Excel file containing RGC identifications, binary matricies, and taxonomic markers. See the “Read-Me” tab for instructions on navigating these data.

## Additional Information and Declarations

### DNA Deposition

The following information was supplied regarding the deposition of DNA sequences: Complete plastomes of Pooideae species sequenced for this study are accessible via GenBank accession numbers MH569074 to MH569089.

### Data Availability

The following information was supplied regarding data availability: Alignment data are available through the TreeBase repository: https://www.treebase.org/treebase-web/search/study/summary.html?id=24067.
