# Supplementary material for: Plastome phylogenomics and characterization of rare genomic changes as taxonomic markers in plastome groups 1 and 2 Poeae (Pooideae; Poaceae)
Source: PeerJ. 2019 Jun 3;7:e6959. doi: 10.7717/peerj.6959 (PMC6553444; doi:10.7717/peerj.6959)
Supplement: Supplemental Information 1 — BI trees for Group 1&2, Group 1, and Group 2 taxa. [file peerj-07-6959-s001.pdf]

SI Figure 1. Majority Consensus Tree for BI Combined Sequence and RGC for Groups 1 & 2.

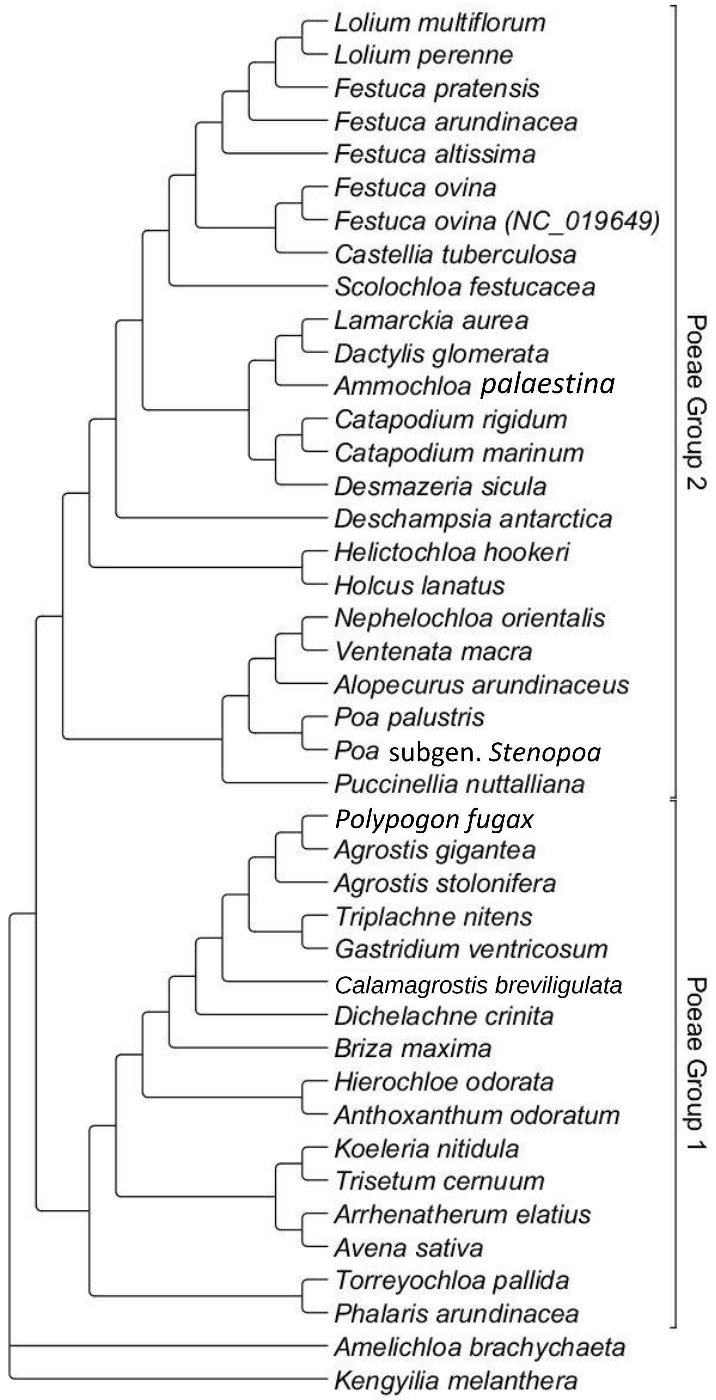

SI Fig. 1. BI combined sequence and RGC data sets of both group 1 and group 2 species produced topologies consistent with sequence only analyses and topologies of previous studies. All nodes at maximum support.

SI Figure 2. Majority Consensus Tree for BI Sequence Analysis Group 1 only.

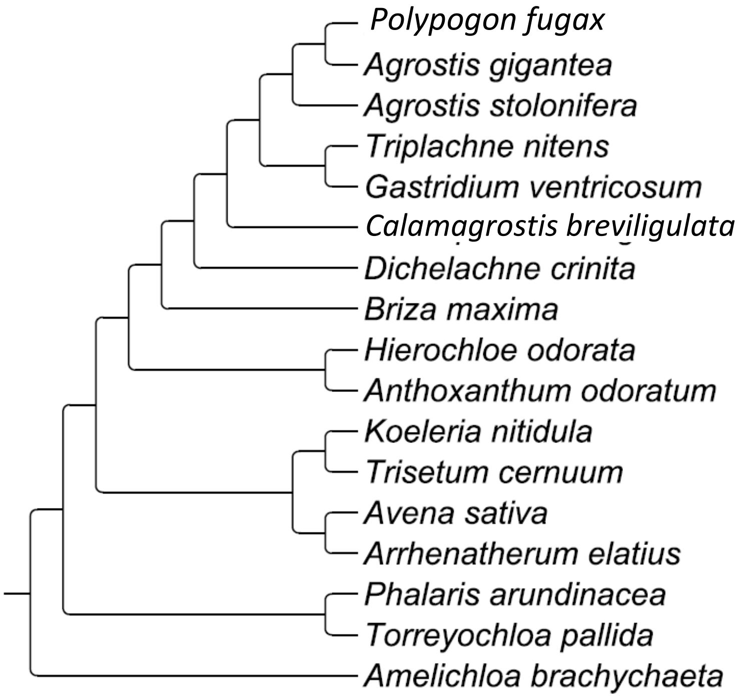

SI Fig. 2. BI sequence only data set of group 1 species produced topologies consistent with sequence and RGC analyses and topologies of previous studies. All nodes at maximum support.

SI Figure 3. Majority Consensus Tree for BI Sequence Analysis for Group 2 only.

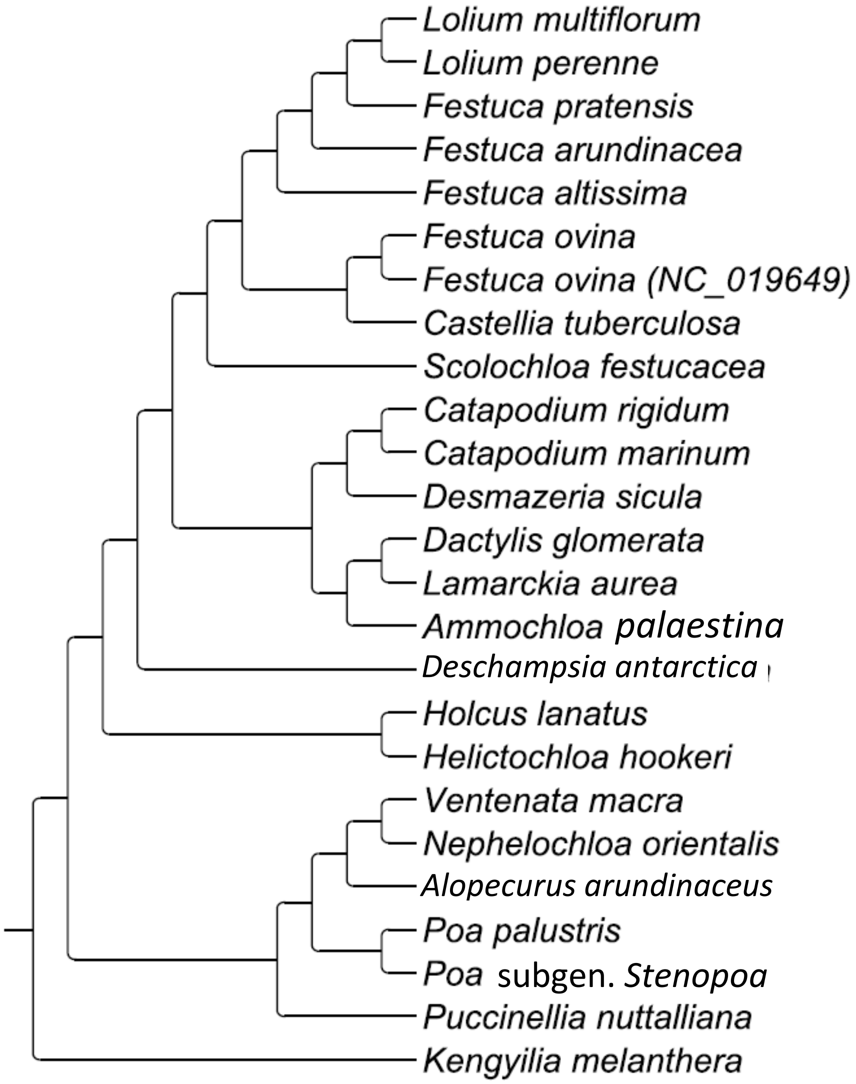

SI Fig. 3. BI sequence only data set of group 2 species produced topologies consistent with sequence and RGC analyses and topologies of previous studies. All nodes at maximum support.
