# Supplementary material for: Plastome phylogenomics and characterization of rare genomic changes as taxonomic markers in plastome groups 1 and 2 Poeae (Pooideae; Poaceae)
Source: PeerJ. 2019 Jun 3;7:e6959. doi: 10.7717/peerj.6959 (PMC6553444; doi:10.7717/peerj.6959)
Supplement: Table S1 — SI.T.1 Data describing ML and BI analyses and resulting phylogenomic trees. G1, Group 1; G2, Group 2; RGC, Rare Genomic Changes. [file peerj-07-6959-s002.pdf]

SI Table 1. Additional data describing ML and BI analyses conducted.

	Mean support value	Std. Dev. bs(ML) | stdev. splfr (BI)	-lnL
**ML**			
Sequence			
G1	99.07	0.84	-223665.52
G2	98.36	0.16	-269023.15
G1+G2	99.10	1.72	-290961.06
Combined			
G1+RGC	99.07	0.82	-223882.83
G2+RGC	98.40	0.27	-269358.13
G1+G2+RGC	91.88	1.79	-291612.57
**BI**			
Sequence			
G1	1.0	0.010476	-227764.13
G2	1.0	0.012298	-275775.33
G1+G2	0.99	0.002266	-302944.15
Combined			
G1+RGC	0.97	0.007857	-230513.31
G2+RGC	1.0	0.00739	-276119.53
G1+G2+RGC	1.0	0.011224	-303590.55

SI.T.1 Data describing ML and BI analyses and resulting phylogenomic trees.

G1 = Group 1; G2 = Group 2; RGC = Rare Genomic Changes.
